# Evaluation of Depression Levels in Patients With Primary Open-Angle Glaucoma Using the Geriatric Depression Scale

**DOI:** 10.7759/cureus.64162

**Published:** 2024-07-09

**Authors:** Ayşegül Barak Özer, Pınar Eröz

**Affiliations:** 1 Psychiatry, Niğde Ömer Halisdemir University Training and Research Hospital, Niğde, TUR; 2 Ophthalmology, Tarsus State Hospital, Mersin, TUR

**Keywords:** visual field defect, low vision, age and depression, depression in chronic illness, primary open angle glaucoma

## Abstract

Introduction

Glaucoma is a chronic disease that can lead to severe visual impairment and blindness.

Methods

The study included 91 primary open-angle glaucoma patients aged 60 years and older (group 1) and 83 healthy controls (group 2) with similar age and gender distribution. The duration of the disease, the number of anti-glaucomatous drops used daily, and visual field parameters were recorded. All participants then underwent a comprehensive mental status examination by a psychiatrist and were administered the Geriatric Depression Scale (GDS).

Results

In Group 1, the mean duration of glaucoma was 10.2±6.0 years, and the mean number of drops used per day was 2.91±1.47 drops/day. According to visual field (24-2) data, the mean mean deviation (MD) was -7.76±4.78 dB and the mean pattern standard deviation (PSD) was 5.14±2.60 dB. According to the classification based on MD, 33 (36.3%) patients were in the early stage, 36 (39.5%) in the intermediate stage, and 22 (24.2%) in the advanced stage. The mean best-corrected visual acuity (BCVA) was 0.85±0.38 logMAR in group 1 and 0.34±0.19 logMAR in group 2. The mean GDS scores were 13.7±7.23 points in group 1 and 3.61±1.71 points in group 2. There were statistically significant differences between the groups in terms of BCVA and GDS scores (p=0.039 and p<0.001, respectively).

Conclusion

In conclusion, it is important that ophthalmologists provide adequate information about glaucoma to prevent the development of depression in patients with glaucoma. This information may protect patients from uncertainty. A multidisciplinary approach in the management of glaucoma, a chronic and vision-threatening disease, can positively affect patients' compliance with follow-up and treatment, increase the quality of healthcare, and improve treatment responses.

## Introduction

Glaucoma is a chronic disease that can lead to severe visual impairment and blindness. It is estimated that up to 80 million people are affected by primary open-angle glaucoma [1]. With an increasing proportion of the elderly population worldwide, glaucoma is placing a growing burden on healthcare services [2]. Depression is considered a global disease by the WHO and is estimated to affect more than 264 million people [3]. The common link between glaucoma and psychiatric disorders is thought to be a neurodegenerative pathway, and patients with glaucoma have an approximately 10-fold higher prevalence of depression than the general population [4,5].

The aim of this study was to evaluate the level of depression in patients with primary open-angle glaucoma.

## Materials and methods

Written informed consent was obtained from all participants. The necessary permissions before the study were obtained from Toros University Scientific Research and Publication Ethics Committee (2024/92). The study was conducted in accordance with the Declaration of Helsinki.

Patients were followed up between December 1, 2023, and April 1, 2024, in the Ophthalmology Clinic for primary open-angle glaucoma. The control group consisted of healthy subjects who were admitted to the clinic on the same dates. The study included 91 patients aged 60 years and older with primary open-angle glaucoma who were followed up in the ophthalmology clinic, and 83 healthy controls with a similar age and gender distribution. Patients who were taking antidepressant medication and/or were being followed up and treated for major depression in a psychiatric clinic were excluded.

Age, gender, and best-corrected visual acuity (BCVA) data were recorded for all participants. Duration of disease, number of anti-glaucomatous drops used daily, and visual field parameters (24-2) data were noted. All participants then underwent a comprehensive mental status examination by a psychiatrist, and the Geriatric Depression Scale (GDS) was administered.

The GDS is a 30-item scale that can be used to assess depression, and its Turkish validity and reliability study was conducted by Ertan and Eren. On the scale, 0 to 10 points are classified as 'no depression,' 11 to 13 points as 'probably depression,' and 14 points or more as 'definite depression' [6].

Statistical analysis of the study data was performed with the SPSS v25.0.1 package program (IBM Corp, Armonk, NY, USA). Categorical variables were expressed as number (n) and percentage (%), and numerical variables were expressed as mean ± SD. The conformity of continuous variables to normal distribution was checked by the Shapiro-Wilk test. The t-test was used to compare the means of the groups. The relationship between categorical variables was investigated by chi-square analysis. The statistical significance level was set at p<0.05 for all comparisons.

## Results

The study included 91 patients (group 1) and 83 healthy controls (group 2). The mean age was 66.0±10.7 years in group 1 and 68.3±6.4 years in group 2. The number of females was 49 (53.8%) in group 1 and 43 (51.8%) in group 2. There was no statistically significant difference between the groups in terms of age and gender (p=0.480 and p=0.788, respectively).

In Group 1, the mean duration of the disease was 10.2±6.0 years, and the mean number of drops used per day was 2.91±1.47 drops/day. According to visual field (24-2) data, the mean deviation (MD) was -7.76±4.78 dB, and the mean pattern standard deviation (PSD) was 5.14±2.60 dB. According to the classification by MD, 33 (36.3%) patients were in the early stage, 36 (39.5%) in the intermediate stage, and 22 (24.2%) in the advanced stages. The mean BCVA was 0.85±0.38 logMAR in group 1 and 0.34±0.19 logMAR in group 2. The mean GDS scores were 13.7±7.23 points in group 1 and 3.61±1.71 points in group 2. There were statistically significant differences between the groups in terms of BCVA and GDS scores (p=0.039 and p<0.001, respectively) (Table 1).

**Table 1:** Participants' study data and Geriatric Depression Scale scores. Numerical variables with mean and standard deviation (SD). Categorical variables (number (n) and percentage (%)). p<0.05 was considered statistically significant. p: Significance value; GDS: Geriatric Depression Scale MD: Mean deviation; PSD: Pattern standard deviation; BCVA: Best-corrected visual acuity.

	Patient	Geriatric Depression Scale (points)				p
	Overall	0 to 10	11 to 13	14 and above	Control	
N	91	30	13	48	83	
Age (years)	66.0±10.7	65.9±9.32	69.2±12.9	65.2±10.9	68.3±6.4	0.480
Female (n,%)	49 (53.8)	19 (63.3)	5 (38.5)	25 (52.1)	43 (51.8)	0.788
Male (n,%)	42 (46.2)	11 (36.7)	8 (61.5)	23 (47.9)	40 (48.2)	
Duration (years)	10.2±6.0	9.9±6.1	11.5±5.2	10.1±6.2	˗	0.694
Topical drops (per day)	2.91±1.47	2.77±1.55	3.15±1.28	2.92±1.49	˗	0.730
MD 24-2 (dB)	-7.76±4.78	-7.87±3.81	-8.15±5.18	-7.58±5.27	˗	0.921
–0.01 to –6.00 dB (n,%)	33 (36.3)	8 (26.7)	5 (38.5)	20 (41.6)		0.207
–6.01 to –12.00 dB (n,%)	36 (39.5)	17 (56.7)	5 (38.5)	14 (29.2)		
–12.01 or worse (n,%)	22 (24.2)	5 (16.6)	3 (23.0)	14 (29.2)		
PSD 24-2 (dB)	5.14±2.60	4.93±2.60	4.62±2.10	5.42±2.74	˗	0.538
BCVA (logMAR)	0.85±0.38	0.76±0.35	0.90±0.37	0.88±0.39	0.34±0.19	0.039
GDS	13.7±7.23	5.20±3.00	11.92±2.36	19.6±3.32	3.61±1.71	<0.001

Correlation analysis revealed statistically significant correlations between GDS scores and the duration of disease (positively very strong, r=0.843), MD value (negatively very strong, r=-0.863), and BCVA (positively moderate, r=0.534) (p=0.021, p=0.018, and p=0.012, respectively) (Table 2).

**Table 2:** Correlation analysis between study parameters and Geriatric Depression Scale scores. r: Correlation coefficient; p: Significance value; GDS: Geriatric Depression Scale MD: Mean deviation; PSD: Pattern standard deviation; BCVA: Best-corrected visual acuity. p<0.05 was considered statistically significant.

		GDS
Age (years)	r	-0.032
	p	0.763
Duration (years)	r	0.843
	p	0.021
Topical drops (per day)	r	0.089
	p	0.404
MD 24-2 (dB)	r	-0.863
	p	0.018
PSD 24-2 (dB)	r	0.361
	p	0.565
BCVA (logMAR)	r	0.534
	p	0.012

## Discussion

The first novelty of the study is the comparison of depression levels in patients according to visual field parameters. Comparisons were also made based on the severity of the disease and the number of medications used by the patients.

The number of patients with glaucoma worldwide is estimated to increase to 111 million by 2040. The prevalence of glaucoma in the middle-aged and older population is 3.5% [7]. In addition to sociodemographic factors, vision loss is significantly associated with depression [8]. Many studies in the literature have investigated the relationship between glaucoma and depression. One study found that treatment compliance in patients with depression was 38 times lower than in patients without depression [5]. However, the rate of depression in glaucoma patients in these studies varied widely, from 10-40%. This variation may be attributed to the sample variability of the studies and the differences in the geographical regions where the studies were conducted [9-12].

Mental changes in glaucoma patients may result from reduced vision but may also stem from potential future functional decline (glaucomatous progression) [13]. Patients who had difficulty with daily activities such as near reading, working, walking down stairs, and daytime driving were significantly more likely to be depressed than those who did not. However, none of the objective measures of glaucoma severity or visual function, including BCVA, presence and severity of visual field defects, number of topical anti-glaucomatous drops, and use of topical beta-blockers, were found to be significant predictors of depression in a multivariable logistic regression model [14].

In depressed patients, neuroplasticity mediated by proteins such as brain-derived neurotrophic factor (BDNF) is impaired [15,16]. Low levels of BDNF may negatively affect the survival of retinal ganglion cells [1]. It is thought that the loss of retinal ganglion cells due to glaucoma progression may be a factor that disrupts light perception, which may affect circadian rhythm, sleep, and mood, potentially impacting psychiatric status [1,17]. One study has shown that the progressive loss of retinal ganglion cells is positively associated with the depression score and that this association is strengthened when the global loss of retinal ganglion cells exceeds a threshold of 15% [18].

The effect of the clinical use of beta blockers on depression is controversial [19,20]. A multicenter study concluded that topical eye drops containing beta-blockers may enter the systemic circulation and cause depression and psychosis-like central nervous system effects, although rarely [21]. In another study, it was observed that the prevalence of depression did not differ significantly between glaucoma patients with and without beta-blocker topical eye drops [11].

Correlation analysis revealed statistically significant correlations between GDS scores and the duration of the disease (positively very strong, r=0.843), MD value (negatively very strong, r=-0.863), and BCVA (positively moderate, r=0.534). This suggests that an increasing duration of the disease, advanced stages, and decreased visual acuity increase depression scores in patients. Chronic diseases can progress over time and cause complications. Moreover, visual loss due to primary open-angle glaucoma can be permanent. Considering all these factors, preventing complications may prevent the development of depression in patients. Additionally, it may increase treatment compliance and improve treatment success.

The limitations of the study include the fact that the evaluation was performed with a relatively small number of patients. Additionally, the effects of patients' depression levels on treatment compliance and prognosis were not evaluated, which is another limitation. Finally, the cross-sectional design does not explain the causes of depression in patients.

## Conclusions

In conclusion, chronic diseases that can permanently reduce vision, such as primary open-angle glaucoma, can affect patients' mental health. Long-term hospitalization for follow-up and/or treatment, the need for daily medication, and potential complications can cause stress, reduce independence, lead to social isolation, and undermine self-confidence. All these factors can contribute to the development of depression in patients. To prevent the development of depression, it is important for family physicians and ophthalmologists to provide patients with adequate information about primary open-angle glaucoma and its progression. This information may protect patients from uncertainty and/or the need for seeking additional treatment. A multidisciplinary approach to the management of primary open-angle glaucoma, involving an ophthalmologist, psychiatrist, physician, and nurse, can positively affect patient compliance with follow-up and treatment, improve the quality of health care, and enhance the response to treatment.
